# Supplementary material for: Geographical risk pattern and temporal trends in incidence of HPV-related cancers in northern Thailand: A population-based study
Source: PLoS One. 2022 Jun 28;17(6):e0270670. doi: 10.1371/journal.pone.0270670 (PMC9239466; doi:10.1371/journal.pone.0270670)
Supplement: S1 Table — (DOCX) [file pone.0270670.s001.docx]

S1 Table data quality indicators.

| Cancers | n (%) | DCO% | Number of morphologically verified (MV%) | | | | | | | | |
| --- | --- | --- | --- | --- | --- | --- | --- | --- | --- | --- | --- |
|  |  |  | Chiang Mai | Lamphun | Lampang | Phrae | Phayao | Chiang Rai | Nan | Mae Hong Son | All northern  Thailand |
| 2008-2012 | 4,448 |  |  |  |  |  |  |  |  |  |  |
| Anal | 104 (2.3%) | 5 | 32 (97) | 6 (100) | 12 (80) | 5 (62) | 14 (88) | 11 (42) |  |  | 80 (77) |
| Cervical | 3,930 (88.4%) | 2 | 1,187 (97) | 271 (71) | 323 (88) | 262 (70) | 267 (69) | 698 (58) |  |  | 3,008 (76) |
| Oropharynx | 52 (1.2%) | 0 | 21 (100) | 3 (75) | 5 (62) | 2 (100) | 4 (80) | 4 (33) |  |  | 39 (75) |
| Penile | 205 (4.5%) | 0.5 | 62 (95) | 16 (73) | 35 (100) | 15 (88) | 11 (52) | 25 (56) |  |  | 158 (79) |
| Vaginal | 48(1.1%) | 0 | 10 (83) | 5 (100) | 3 (100) | 4 (100) | 6 (86) | 16 (89) |  |  | 44 (90) |
| Vulvar | 109 (2.4%) | 0 | 38 (100) | 2 (67) | 7 (88) | 10 (100) | 11 (85) | 30 (81) |  |  | 98 (90) |
| 2013-2017 | 3,883 |  |  |  |  |  |  |  |  |  |  |
| Anal | 141(3.6%) | 1 | 45 (98) | 12 (92) | 19 (83) | 8 (80) | 6 (86) | 21 (78) | 9 (90) | 4 (80) | 124 (88) |
| Cervical | 3,229 (83.2%) | 1 | 911 (97) | 231 (86) | 358 (92) | 221 (80) | 234 (80) | 642 (80) | 171 (90) | 60 (82) | 2,828 (88) |
| Oropharynx | 86 (2.2%) | 1 | 33 (100) | 8 (100) | 9 (90) | 5 (83) | 2 (100) | 14 (74) | 1 (33) | 5 (100) | 77 (90) |
| Penile | 224 (5.8%) | 0 | 75 (99) | 11 (77) | 34 (97) | 12 (92) | 14 (82) | 26 (77) | 20 (95) | 11 (79) | 203 (91) |
| Vaginal | 50 (1.3%) | 2 | 3 (75) | 6 (100) | 5 (100) | 7 (100) | 9 (82) | 12 (80) | 2 (100) | NA | 44 (88) |
| Vulvar | 153 (3.9%) | 1 | 54 (96) | 10 (77) | 17 (100) | 9 (90) | 15 (94) | 27 (82) | 3 (75) | 4 (100) | 139 (91) |
