# Supplementary material for: Geographical risk pattern and temporal trends in incidence of HPV-related cancers in northern Thailand: A population-based study
Source: PLoS One. 2022 Jun 28;17(6):e0270670. doi: 10.1371/journal.pone.0270670 (PMC9239466; doi:10.1371/journal.pone.0270670)
Supplement: S2 Table — (DOCX) [file pone.0270670.s002.docx]

S2 Table. Areas with relative risk (RR) significantly higher than 1 by cancer type, 2013-2017.

| Cancer | Districts | Province | RR | 95%CrI |
| --- | --- | --- | --- | --- |
| Cervical cancer | 43.Wiang Haeng | Chiang Mai | 2.23 | 2.13-2.34 |
|  | 11. Mae Sai | Chiang Rai | 1.85 | 1.77-1.95 |
|  | 35. Fang | Chiang Mai | 1.47 | 1.4-1.55 |
|  | 2.Khun Tan | Chiang Rai | 1.41 | 1.34-1.48 |
|  | 31.Chai Prakan | Chiang Mai | 1.33 | 1.27-1.40 |
|  | 14.Wiang Pa Pao | Chiang Rai | 1.19 | 1.13-1.24 |
|  | 30.Chiang Dao | Chiang Mai | 1.17 | 1.12-1.23 |
|  | 4. Chiang Saen | Chiang Rai | 1.17 | 1.12-1.23 |
|  | 41.Mae Ai | Chiang Mai | 1.17 | 1.11-1.22 |
|  | 75.Den Chai | Phrae | 1.12 | 1.07-1.18 |
|  | 5.Thoeng | Chiang Rai | 1.11 | 1.06-1.16 |
|  | 7.Phan | Chiang Rai | 1.11 | 1.05-1.16 |
|  | 8.Mae Chan | Chiang Rai | 1.10 | 1.05-1.16 |
|  | 99.Pai | Mae Hong Son | 1.07 | 1.02-1.12 |
|  | 20. Chun | Phayao | 1.06 | 1.01-1.11 |
|  | 9. Mae Fa Luang | Chiang Rai | 1.06 | 1.01-1.11 |
|  | 55.Ban Hong | Lamphun | 1.05 | 1.00-1.11 |
| Oropharyngeal | 28. Mueang Chiang Mai | Chiang Mai | 1.340 | 1.02-1.71 |
| Anal | 60.Wiang Nong Long | Lamphun | 1.260 | 1.03-1.51 |
|  | 38.Mae Taeng | Chiang Mai | 1.240 | 1.02-1.49 |
| Penile | 11. Mae Sai | Chiang Rai | 8.63 | 7.31-10.07 |
|  | 9. Mae Fa Luang | Chiang Rai | 3.04 | 2.57-3.55 |
|  | 103. Pang Mapha | Mae Hong Son | 2.01 | 1.70-2.34 |
|  | 50. Omkoi | Chiang Mai | 1.82 | 1.54-2.12 |
|  | 47. San Pa Tong | Chiang Mai | 1.66 | 1.41-1.94 |
|  | 36.Phrao | Chiang Mai | 1.66 | 1.41-1.94 |
|  | 68. Mueang Pan | Lampang | 1.63 | 1.38-1.90 |
|  | 43.Wiang Haeng | Chiang Mai | 1.62 | 1.37-1.89 |
|  | 97. Mueang Mae Hong Son | Mae Hong Son | 1.58 | 1.34-1.85 |
|  | 40. Mae Wang | Chiang Mai | 1.53 | 1.29-1.78 |
|  | 41.Mae Ai | Chiang Mai | 1.42 | 1.20-1.65 |
|  | 102.Sop Moei | Mae Hong Son | 1.41 | 1.19-1.64 |
|  | 31.Chai Prakan | Chiang Mai | 1.36 | 1.15-1.58 |
|  | 49.Hang Dong | Chiang Mai | 1.32 | 1.11-1.54 |
|  | 8.Mae Chan | Chiang Rai | 1.27 | 1.08-1.48 |
|  | 35.Fang | Chiang Mai | 1.26 | 1.07-1.47 |
|  | 100. Mae Sariang | Mae Hong Son | 1.26 | 1.07-1.47 |
|  | 101. Mae La Noi | Mae Hong Son | 1.22 | 1.03-1.42 |
| Vaginal | 26. Phu Sang | Phayao | 2.70 | 1.78-3.84 |
|  | 21. Chiang Kham | Phayao | 2.12 | 1.40-3.01 |
|  | 17. Doi Luang | Chiang Rai | 1.82 | 1.20-2.59 |
|  | 5.Thoeng | Chiang Rai | 1.56 | 1.03-2.22 |
| Vulvar | 35.Fang | Chiang Mai | 1.73 | 1.42-2.09 |
|  | 30. Chiang Dao | Chiang Mai | 1.52 | 1.24-1.83 |
|  | 31.Chai Prakan | Chiang Mai | 1.35 | 1.10-1.62 |
|  | 7.Phan | Chiang Rai | 1.29 | 1.05-1.55 |
