# Supplementary material for: Geographical risk pattern and temporal trends in incidence of HPV-related cancers in northern Thailand: A population-based study
Source: PLoS One. 2022 Jun 28;17(6):e0270670. doi: 10.1371/journal.pone.0270670 (PMC9239466; doi:10.1371/journal.pone.0270670)
Supplement: S3 Table — (DOCX) [file pone.0270670.s003.docx]

S3 Table. Areas with relative risk (RR) significantly higher than 1 for cervical cancer by time period.

| Period | Districts | Province | RR | 95%CrI |
| --- | --- | --- | --- | --- |
| 2008-2012 | Pa Daet | Chiang Mai | 1.60 | 1.53-1.68 |
|  | Mae Sai | Chiang Rai | 1.53 | 1.46-1.61 |
|  | Mae Suai | Chiang Rai | 1.45 | 1.39-1.52 |
|  | Mae Chan | Chiang Rai | 1.44 | 1.38-1.51 |
|  | Fang | Chiang Mai | 1.42 | 1.35-1.49 |
|  | Wiang Chai | Chiang Rai | 1.42 | 1.35-1.48 |
|  | Wiang Haeng | Chiang Mai | 1.41 | 1.34-1.48 |
|  | Mae Fa Luang | Chiang Rai | 1.36 | 1.3-1.43 |
|  | Ban Hong | Lamphun | 1.34 | 1.27-1.40 |
|  | Dok Khamtai | Phayao | 1.31 | 1.25-1.39 |
|  | Mae Ai | Chiang Mai | 1.31 | 1.24-1.37 |
|  | Wiang Pa Pao | Chiang Rai | 1.29 | 1.23-1.36 |
|  | Mueang Chiang Rai | Chiang Rai | 1.25 | 1.19-1.31 |
|  | Chiang Muan | Phayao | 1.25 | 1.19-1.31 |
|  | Chiang Dao | Chiang Mai | 1.22 | 1.17-1.28 |
|  | Chiang Khong | Chiang Rai | 1.17 | 1.12-1.23 |
|  | Mae Lao | Chiang Rai | 1.13 | 1.07-1.18 |
|  | Den Chai | Phrae | 1.12 | 1.07-1.18 |
|  | Mae On | Chiang Mai | 1.11 | 1.05-1.16 |
|  | Mueang Lamphun | Lamphun | 1.10 | 1.05-1.15 |
|  | San Pa Tong | Chiang Mai | 1.07 | 1.02-1.12 |
| 2013-2017 | Wiang Haeng | Chiang Mai | 2.52 | 2.4-2.64 |
|  | Mae Sai | Chiang Rai | 1.86 | 1.77-1.95 |
|  | Fang | Chiang Mai | 1.49 | 1.41-1.56 |
|  | Khun Tan | Chiang Rai | 1.41 | 1.34-1.48 |
|  | Chai Prakan | Chiang Mai | 1.34 | 1.27-1.41 |
|  | Mae Ai | Chiang Mai | 1.21 | 1.15-1.27 |
|  | Chiang Dao | Chiang Mai | 1.20 | 1.14-1.26 |
|  | Wiang Pa Pao | Chiang Rai | 1.19 | 1.13-1.25 |
|  | Chiang Saen | Chiang Rai | 1.18 | 1.13-1.24 |
|  | Den Chai | Phrae | 1.13 | 1.07-1.18 |
|  | Thoeng | Chiang Rai | 1.12 | 1.06-1.17 |
|  | Phan | Chiang Rai | 1.11 | 1.06-1.17 |
|  | Mae Chan | Chiang Rai | 1.11 | 1.06-1.17 |
|  | Mae Fa Luang | Chiang Rai | 1.07 | 1.02-1.13 |
|  | Chun | Phayao | 1.07 | 1.02-1.12 |
|  | Wiang Chai | Chiang Rai | 1.06 | 1.01-1.11 |
