# Supplementary material for: Geographical risk pattern and temporal trends in incidence of HPV-related cancers in northern Thailand: A population-based study
Source: PLoS One. 2022 Jun 28;17(6):e0270670. doi: 10.1371/journal.pone.0270670 (PMC9239466; doi:10.1371/journal.pone.0270670)
Supplement: Attachment — Submitted filename: Response to Reviewers.docx [file pone.0270670.s005.docx]

Editor PLOS ONE

May 5, 2022

**RE: Ms. No. PONE-D-22-04051**

Dear Editor,

I would like to thank you for sending the comments of the reviewers following the peer review of our Research Article entitled “Geographical Risk Pattern and Temporal Trends in Incidence of HPV-related Cancers in Northern Thailand: A Population-Based Study”.

Please find attached the revised manuscript. We have addressed the journal requirement, the editor comments and the reviewers’ comments in the order they were provided. All changes are tracked in the manuscript.

Journal requirement:

***Requirement 1:*** *When submitting your revision, we need you to address these additional requirements. Please ensure that your manuscript meets PLOS ONE's style requirements, including those for file naming.*

**Response:** We have corrected the manuscript format to meet PLOS ONE's style.

***Requirement 2:*** *We note that the grant information you provided in the ‘Funding Information’ and ‘Financial Disclosure’ sections do not match.*

**Response:** This research work was supported by Chiang Mai University. There was no grant numbers. We have removed funding statement from the manuscript.

***Requirement 3****. We note that you have indicated that data from this study are available upon request. PLOS only allows data to be available upon request if there are legal or ethical restrictions on sharing data publicly.*

**Response:** We have uploaded the minimal anonymized data as Supporting Information files. Please update the Data Availability statement on our behalf.

***Requirement 4****. Thank you for stating the following in the Acknowledgments Section of your manuscript: (We would like to thank the Bureau of Registration Administration, Ministry of Interior, for providing death certificate data for cancer registry. This research work was supported by Chiang Mai University.) We note that you have provided funding information that is not currently declared in your Funding Statement. However, funding information should not appear in the Acknowledgments section or other areas of your manuscript. We will only publish funding information present in the Funding Statement section of the online submission form.* 

**Response:** We removed funding-related text from the manuscript and simplified our Funding Statement as follows.

“This research work was supported by Chiang Mai University.

Please update the Funding Statement on our behalf. Many thanks.

***Requirement 5*** *We note that Figures 3, 4, 5, and 6 your submission contain map images which may be copyrighted. All PLOS content is published under the Creative Commons Attribution License (CC BY 4.0), which means that the manuscript, images, and Supporting Information files will be freely available online, and any third party is permitted to access, download, copy, distribute, and use these materials in any way, even commercially, with proper attribution. For these reasons, we cannot publish previously copyrighted maps or satellite images created using proprietary data, such as Google software (Google Maps, Street View, and Earth). For more information, see our copyright guidelines:* [*http://journals.plos.org/plosone/s/licenses-and-copyright*](http://journals.plos.org/plosone/s/licenses-and-copyright)*.
 
We require you to either (1) present written permission from the copyright holder to publish these figures specifically under the CC BY 4.0 license, or (2) remove the figures from your submission:*

**Response:** We generated all the maps using a free software, Quantum Geographic Information System program (QGIS). The mapping shapefile in district level in Thailand was freely access from Global Administrative Database (geoBoundaries). We have added the sentences about the software use and source of shape file as follows (Page 6 Line 140-142). We also added references accordingly.

“A map of the incidence patterns was then generated using the Quantum Geographic Information System program version 3.4.15-1 [23]. The shapefile used was from Global Administrative Database (geoBoundaries): an online, open license resource of the geographic boundaries of political administrative divisions [24].”

We also added the software use and the source of shapefile in the figure legends.

***Requirement 6****: Please include captions for your Supporting Information files at the end of your manuscript, and update any in-text citations to match accordingly. Please see our Supporting Information guidelines for more information:* [*http://journals.plos.org/plosone/s/supporting-information*](http://journals.plos.org/plosone/s/supporting-information).

**Response:** We now include captions of Supporting Information files at the end of the manuscript.

***Requirement 7:*** *Please review your reference list to ensure that it is complete and correct. If you have cited papers that have been retracted, please include the rationale for doing so in the manuscript text, or remove these references and replace them with relevant current references. Any changes to the reference list should be mentioned in the rebuttal letter that accompanies your revised manuscript. If you need to cite a retracted article, indicate the article’s retracted status in the References list and also include a citation and full reference for the retraction notice*.

**Response:** We have corrected the reference format to meet the guideline of the journal. We confirmed that there was no retracted publication in the list.

Editor Comments:

***Comment 1****: P5 L117 what was the rationale for selecting those 2 particular time periods? It might be worth highlighting that both are 5 year periods.*

**Response:** This study aims to measure inequality in the risk of incidence of HPV-related cancers in term of spatial risk patterns in the eight provinces, the entire upper northern Thailand in which the data available in the time period of 2013-2017, the latest and the most complete of population-based cancer registry in northern Thailand. For cervical cancer which is still one of common cancer in Thailand in particular in Northern part, we also evaluated the geographical risk pattern in the previous 5-years period (2008-2012) and compared with the later period to describe the change in inequality of the risk of its incidence.

We now clarified this point and we are highlighting that both are 5-years periods as suggested (Page 5 Line 119-124).

***Comment 2****: P5 L120 what is meant by “morphologic verification”? later in the manuscript it appears that this was abbreviated to MV%? If so, please clarify*

**Response:** We have clarified the MV% which is abbreviation of morphologic verification as suggested, Page 6, Line 128.

***Comment 3****: P5 L121 The part about diagnosis from death certificate is also a bit unclear and should be better explained.*

**Response:** As suggested, we have clarified the cases that diagnosis from death certificate only in which the confirmed information cannot be traced back and abbreviated to DCO%, Page 6, Line 130.

***Comment 4****: P5 L128 the methods mention that credible intervals (CrI) were calculated. However, this information is never shown in the manuscript. Consider including the calculated information at least in supplementary tables*

**Response:** We have added the credible intervals (CrI) in the results part and in the supplementary tables as suggested.

S2 Table. Areas with relative risk (RR) significantly higher than 1 by cancer type, 2013-2017.

S3 Table. Areas with relative risk (RR) significantly higher than 1 for cervical cancer by time period.

***Comment 5****: P5 L129 Some reference, version number, or at least a link to the QGIS program should be provided. Are there some specific options used that would allow others to analyze the data in the same way?*

**Response:** We have added the reference of QGIS program as follows.

“23. QGIS Development Team. QGIS Geographic Information System version 3.4.15-1: Open Source Geospatial Foundation Project. 2021 [cited 2022 15 April]. Available from: <http://qgis.osgeo.org>.”

***Comment 6****: P5 L138 the cancer types and numbers provided should be sorted in some meaningful order. Ie HPV involvement, descending numbers, alphabetical order or some other consistent order. The same order should be used in Supplementary Table 1 as well as throughout the manuscript and figures*

**Response:** Thank you for your comment. We are now sort the cancer type by alphabetical order in the results part (Page 6, Line 148-154), Figure 3, and Supplementary Table 1.

***Comment 7****: P6 L148 the sentence “The RR ranges from 1.05 to 2.23” might be factually inconsistent with figure 2 since it is obvious that some regions pictured have RR <1*

**Response:** Indeed, the ranges of RR in this sentence was the range of relative risk that was significantly higher than 1. We agreed with reviewer that this inconsistent with figures and confusing. We now provided this results in S2 Table and referrer to the texts in the manuscript.

***Comment 8****: P6 L149 and 153 (and elsewhere) Was some statistical test performed to compare incidences between study regions or time periods, if so some p value and test used should be shown? If not, the use of “significantly” might be misleading and should be replaced with “**noticeably” or some other similar word which could not be misunderstood for statistical significance.*

**Response:** Thank you for your comment. We have clarified the term of “significantly” that the RR were significantly higher than 1 when the 95% CrI were not cover 1 and used the word “noticeably” to describe the decrease of cervical cancer trends as suggested.

***Comment 9****: Can the underlying data of Figures 1/4 be supplied as supplemental table (and include the same information for other cancer types? Figures 1 and 4 should be combined since they present very similar data, at least as subpanels*

**Response:** Since we focus more in the cervical cancer which is the most common HPV-related cancer while the incidence of other cancers is stable low. The scale of cervical cancer incidence was much higher than the others, we separated the cervical cancer from the other HPV-related cancers. We have added the information for Figure 1 and Figure 4 which are now Figure 2 and Figure 3, respectively.

***Comment 10:*** *Can Figure 2/3/5/6 colour scales be changed so that RR less than one is in shades of green (good) and RR above 1 in shades of red (bad) or some other 2 color scale combination. Currently it is difficult to spot areas with RR~1 and maybe areas with lower RR are not emphasized as such and appear more like a „baseline“ instead of "less than average"*

**Response:** We have modified the color scale as suggested and use the “baseline reference” instead of “the average of the incidence in Northern Thailand” as suggested.

***Comment 11****: Instead of repeatedly listing the names of 103 provinces in each figure 2/3/5/6 please make a supplementary table with all the names and refer to this list from each figure legend. The same supplementary table could/should include some of the underlying data (ie. actual number of cases found for each cancer type as well as the actual calculated RR number, population used for calculation, etc..*

**Response:** Thank you for your comments. We now describe in the material and methods the study area using the map and listing the names of 103 provinces. Since we emphasized the RR that were significantly more than 1, we therefore provided the S2 and S3 Tables to presents the RR for figure 2/3/5/6. We also provided aggregated data used for the analysis (S1 Data) that contains the actual number of cases, expected number of cases and population, data used for calculation.

***Comment 12****:* *Subheadings could be emphasized by bold font to be better distinguishable from main text.*

**Response:** Subheadings is now emphasized by bold font as suggested.

***Comment 13****: First line paragraph indents are inconsistently used (ie. P7 L173 vs 179)*

*The language needs some revision and proofreading with some particular points highlighted below*

**Response:** As suggested, the English proofreading have been done for this revised manuscript by the native English.

***Comment 14****:*

*P3 L62 „cancers cause by viral infections“ should be „**are caused by“*

*P3L83 „and increase survival“ should be „and increased survival“*

*P4 L100 typo “oropharyngeal, virginal, vulvar,“ should be vaginal*

*P5 L138 typo space „48(1.1%)“*

*P9 L226 lack of commas „cervical vaginal vulvar and“*

*P9 L227 grammar „This may relevant to the limited“*

*P9 L233 grammar „some categories Thai citizens“*

**Response:** We have corrected the typo and grammatical errors as suggested.

***Comments of the reviewers:***

Reviewer #1

***Comment 1****: What’s “MV%”, ASR and DCO%? Please define acronyms the first time they appear in the text.*

**Response:** We now define acronyms of “MV%”, “ASR” and “DCO%” as suggested (Page 6, Line 128 and Line 130).

***Comment 2****: The screening strategy using Pap-smear and visual inspection with acetic acid (VIA) has started in 2005. Why separate the data into two time periods as 2008-2012 and 2013-2017?*

**Response:** This study aims to measure inequality in the risk of incidence of HPV-related cancers in term of geographical risk patterns in the eight provinces, the entire upper northern Thailand in which the data available in the time period of 2013-2017, the latest and the most complete of population-based cancer registry in northern Thailand. For cervical cancer which is still one of common cancer in Thailand in particular in Northern part, we also evaluated the geographical risk pattern in the previous 5-years period (2008-2012) and compared with the later period to describe the change in inequality of the risk of its incidence.

We now clarified this point as suggested (Page 5 Line 119-124).

***Comment 3****: In the results, please report calculated 95% credible intervals.*

**Response:** We have added 95% credible intervals in the results part as suggested.

***Comment 4****: In Figures 5 and 6, which time period was presented for the incidences by province?*

**Response:** Thank you for your comment. We have clarified that Figure 5 and 6 show the geographical risk pattern of non-cervical HPV-related cancers in the time period of 2013-2017.

***Comment 5****: Page 169-172, From figure 5, it has no information for sex. Why do you say that the incidence rate of HPV related cancers in “both sexes” were significantly higher than the average incidence in Northern Thailand in a specific district of Lamphun Province?*

**Response:** Thank you for your comment**.** We have removed the words “both sexes” to avoid confusing.

Reviewer #2:

***Comment 1****:* *In my opinion the authors should further stress the concept that* *the attributable risk for HPV related cancer largely varies according to cancer type -it is 100% only for cervical cancer. Therefore any interpretation must take into account the other risk factors -e.g., smoking for oral cavity.*

**Response:** Thank you for your suggestion. We have inserted in the discussion method about this point (Page 13 Line 311-313).

***Comment 2****:*  *The English text needs revision*

**Response:** As suggested, the English proofreading have been done for this revised manuscript by the native English.

Reviewer #3:

***Comment 1****: Title - the use of spatial analysis in the title is a little confusion - I would suggest* *'Geographical and Temporal trends in HPV-related cancers in Northern Thailand' as an alternative*

**Response:** Thank you for your suggestion. We have modified the title as suggested as follows.

“Geographical Risk Pattern and Temporal Trends in Incidence of HPV-related Cancers in Northern Thailand: A Population-Based Study”

***Comment 2****: Results - I would separate out temporal trends in incidence and then geographical. When presenting the temporal trends in the text I think you should be more explicit that there was no increase over time (then present the range). At first glance it appears that you say there is no increase and then present the ASR that appears to rise. The figures help of course.*

**Response:** We now separate out temporal trends in incidence and then geographical as suggested and explicit that there was no increase over time for all non-cervical cancers.

***Comment 3****: Results - Figure of ASR - I would have a single figure with all cancer types (as opposed to cervical cancer separate)*

**Response:** We focus more in the cervical cancer which is the most common among HPV-related cancer. The incidence of other cancers is stable low. The scale of cervical cancer incidence was much higher than the others. We therefore separated the cervical cancer from the other HPV-related cancer while combined all non-cervical cancers as a single figure as suggested.

***Comment 4****: Results - geographical spread figure(s) - these are going to be very difficult to read on the page - suggest pick out the key one or two and put the rest in supplementary material.*

**Response:** Thank you for your comment. We agreed with the reviewer that geographical figures were very difficult to read. However, the main objective of this study is to described geographical risk pattern of HPV-related cancer. To simplified the geographical figures, we now describe in the material and methods the study area using the map and listing the names of 103 districts and the remove the district list from the figures.

***Comment 5****:* *Discussion - para 3 - sentence ' This may relevant to the limited to the domain....' this is poorly written and would be better simplified e.g. '**This may be related to documented challenges in transnational mobility and associated health care access'.*

**Response:** We have corrected as suggested.

Best regards,

Kriengkrai Srithanaviboonchai on behalf of all authors
